# The relative effects of upwelling and river flow on the phytoplankton diversity patterns in the ria of A Coruña (NW Spain)

**DOI:** 10.1007/s00227-017-3126-9

**Published:** 2017-03-30

**Authors:** Antonio Bode, Manuel Varela, Ricardo Prego, Fernando Rozada, Martin D. Santos

**Affiliations:** 1 Instituto Español de Oceanografía, Centro Oceanográfico de A Coruña, Apdo, 130, 15080 A Coruña, Spain; 2 Instituto de Investigaciones Marinas, CSIC, Eduardo Cabello, 6, 36208 Vigo, Spain; 3 Instituto de Ganadería de Montaña, CSIC-Universidad de León, Finca Marzanas, Ctra. Grulleros, 24346 León, Spain

**Keywords:** Connectivity, Phytoplankton community, Diversity, Upwelling, Estuary, Freshwater flow

## Abstract

Phytoplankton species assemblages in estuaries are connected to those in rivers and marine environments by local hydrodynamics leading to a continuous flow of taxa. This study revealed differential effects of upwelling and river flow on phytoplankton communities observed in 2011 along a salinity gradient from a river reservoir connected to the sea through a ria-marine bay system in A Coruña (NW Spain, 43° 16–21′ N, 8° 16–22′ W). With 130 phytoplankton taxa identified, the assemblages were dominated in general by diatoms, particularly abundant in the bay and in the estuary, but also by chlorophycea and cyanobacteria in the reservoir. Considering the entire seasonal cycle, the local assemblages were mainly characterized by changes in cryptophytes and diatoms, small dinoflagellates and some freshwater chlorophycea. Salinity, nitrate, and organic matter variables, were the main environmental factors related to the changes in the phytoplankton communities through the system, while phosphate and nitrite were also important for local communities in the estuary and the bay, respectively. The corresponding local phytoplankton assemblages showed moderate levels of connectivity. The estuarine community shared a variable number of taxa with the adjacent zones, depending on the relative strength of upwelling (major influence from the bay) and river flow (major influence of the reservoir) but had on average 35% of unique taxa. Consequently, local and zonal diversity patterns varied seasonally and were not simply related to the salinity gradient driven by the river flow.

**Electronic supplementary material:**

The online version of this article (doi:10.1007/s00227-017-3126-9) contains supplementary material, which is available to authorized users.

## Introduction

Estuarine phytoplankton is typically under the influence of river and marine fluxes. Gradual changes in salinity and temperature caused by changes in these fluxes are recognized as the main environmental factors affecting phytoplankton composition (Olli et al. 2015). Both freshwater and marine species are transported to the estuary where they must survive in their salinity tolerance limits. In addition, biological processes such as grazing are also able to modify estuarine phytoplankton communities with alterations propagated through the food web at multiple temporal and spatial scales (Cloern and Dufford 2005; Lucas et al. 2016). The influence of spatial and temporal scaling in estuarine ecosystems is larger than for any other aquatic system. Connectivity across the various scales and within the water body is provided by the hydrodynamics and they are characterized by one or more ecotones (Elliot and Whitfield 2011). The transitional characteristic of estuarine water connects phytoplankton communities from rivers and marine coastal habitats, where changes in species composition were mainly driven by the balance between freshwater and marine inputs (Muylaert et al. 2009; Dorado et al. 2015). This feature has major implications for phytoplankton diversity. First by increasing the total number of species expected when considering the whole salinity gradient and including all the connected local habitats. Second, by decreasing the local diversity in the different habitats created by the salinity (and other environmental factors) as only a subset of species is adapted to survive in a particular combination of environmental conditions. Finally, by allowing a continuous flow of species to the estuary and marine coastal habitats that may affect the persistence of populations in the context of environmental fluctuations (Aiken and Navarrete 2011).

Connectivity of habitats in space and time implies the migrations of species or their reproductive products, such as spores and eggs (McKinnon et al. 2015), but has also large implications in ecosystem properties affecting nutrient availability, productivity and food web processes (Cloern and Jassby 2010). In well-mixed estuaries (for instance when tides are strong) a homogenization of plankton populations is expected in the marine-influenced habitats, thus enhancing population connectivity by symmetric dispersal. Intense mixing will allow the coexistence of species even when some of them are not fully adapted to local conditions, at least at short timescales when biological interactions (e.g., competition or grazing) are relatively less important than physical transport (Leibold and Norberg 2004). Such mixing would affect diversity by increasing the local component and decreasing between-habitat diversity. In addition, the flow of a dominant current (as the river flow or the currents induced by coastal upwelling) can be considered as a mechanism of asymmetric dispersal in the brackish water domain. This flow will increase the probability of persistence of the populations when facing large environmental perturbations (Aiken and Navarrete 2011).

In the coast of Galicia (NW Spain), most estuaries are included in larger hydrological systems or rias composed by the river, a marine embayment and the adjacent shelf (Alvarez-Salgado et al. 2000; Prego 2002). The flow of the rivers is generally slow, compared to the major flow of seawater driven by tides and the seasonal upwelling (Barton et al. 2015). Upwelling is driven by northerly winds with maximum intensity and frequency between March and October and forces shelf water into the rias, where the input of nutrients is effectively translated in an increase in phytoplankton growth rates. The export of surface water from the rias to the adjacent shelf and subsequent reinjection of coastal water with remineralized nutrients into the rias by repeated upwelling events greatly amplifies the phytoplankton productivity cycle and sustains high biomass of plankton and benthos in this region (Figueiras et al. 2002). In contrast, southerly winds, more frequent between November and February, produce downwelling conditions and accumulate warm, low salinity and less dense shelf surface waters towards the coast and inside the rias (Alvarez-Salgado et al. 2000; Gómez-Gesteira et al. 2003). The upwelling affects large spatial areas and water turnover inside the rias. Typically, wind-driven alongshore flow dominates in the marine domain of the ria, while the downwelling–upwelling cycle is the main driver of the circulation in the middle areas, and tides and freshwater inputs determine water exchanges in the inner, estuarine zone (Barton et al. 2015). In addition, the flow of the rivers contributing to most rias is regulated by the operation of reservoirs for urban and industrial freshwater supply (Gómez-Gesteira et al. 2003; Gago et al. 2005). Upwelling has a major impact on the composition of phytoplankton communities in some of the rias where the exchanges with the shelf are favored by a geographical orientation parallel to the dominant wind flow (e.g., Varela et al. 2004) and lower impact on rias with other orientations (e.g., Bode et al. 2005). In the latter rias, the interactions between upwelling conditions and freshwater inputs greatly affect the species composition (Varela et al. 2001, 2005).

While most studies of phytoplankton communities in estuaries and rias focus on the description of the assemblages as a result of appropriate combinations of environmental variables (e.g., Figueiras and Pazos 1991; Figueiras et al. 2002; Varela and Prego 2003; Brito et al. 2014; Sin and Jeong 2015), in this study we focus on changes in diversity patterns to address the differential role of the major drivers of water exchange on the taxonomical composition of phytoplankton. This approach was applied to a system composed of a marine bay affected by coastal upwelling and downwelling processes, an estuary, and a river regulated by a reservoir. The objective is to analyze the differential effects of upwelling intensity and river flow on the similarity and diversity of phytoplankton communities along the salinity gradient in the coast of A Coruña (Galicia, Spain).

## Materials and methods

### Study area

**Fig. 1:**
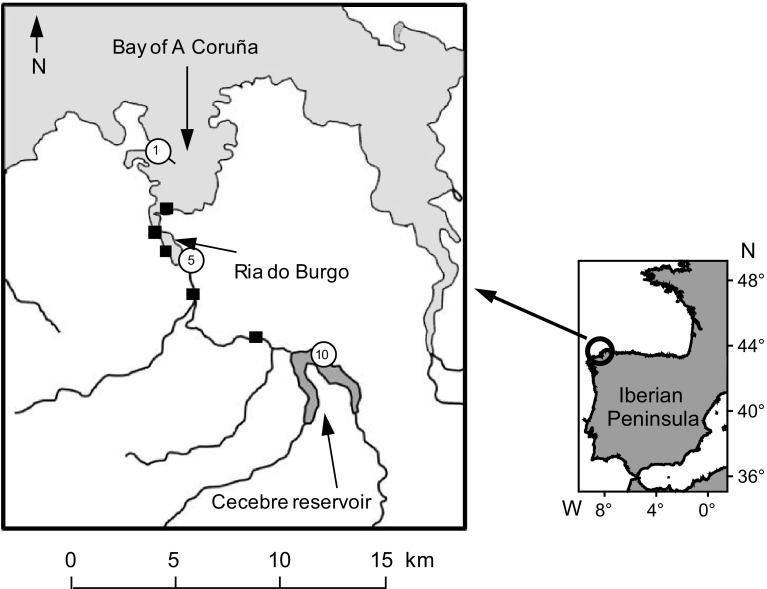
Map of the study area with location of sampling stations during 2011. *Numbered circles* indicate stations where phytoplankton species abundance was studied. 1: bay, 2: estuary, 3: reservoir

The study was conducted in the ria of A Coruña, a complex system characterized by a salinity gradient provided by the river Mero, the estuary of Ria do Burgo and the bay of A Coruña (Fig. 1). The river Mero is the main freshwater contributor to the estuary and its flow is regulated by the Cecebre reservoir which provides water supply for A Coruña and nearby urban areas. The river Mero and its tributaries have a catchment area of 345 km^2^ and a main channel 46 km long before reaching the Ria do Burgo. With 70% of the total flow obtained from precipitation reaching the estuary, this river basin has relatively little seepage and runoff. It has a mean annual flow of 6.6 m^3^ s^−1^, with the high water period between December and March, and the minimum in September (Gómez-Gesteira et al. 1999). Because of the Cecebre reservoir the flow of the river into the ria can be adjusted independently of precipitation. The Ria do Burgo, with a total length of 4 km and an average depth of 2 m, has the characteristics of a tidal estuary where the influence of the river Mero is detected at the surface by the salinity gradient (González 1975; Gómez-Gesteira et al. 1999). The Bay of A Coruña, with an average depth of 25 m, an area of 24 km^2^ and a mouth of 3 km, is characterized by a dominant marine influence (Fraga 1996; Varela et al. 2001; Varela and Prego 2003).

### Physical and chemical properties of water

Ten sampling campaigns were carried out, at approximately monthly frequency between February and December 2011. Samples were collected during the flood tide from eight stations distributed along the salinity gradient between the Cecebre reservoir and the internal side of the breakwater of the port of A Coruña (Fig. 1). Surface water was collected from the existing bridges or from the shore using a 10-L acid-washed polycarbonate container equipped with a polyester rope of 5 m. Subsamples for determination of particulate and dissolved substances were collected from the container and stored according to the specific analyses.

Water temperature (*t*) and salinity (Sal) were measured with a probe (YSI Model 30). Salinity measurements were given according to the Practical Salinity Scale (UNESCO 1984). Concentration of inorganic nutrients (nitrate, nitrite, phosphate, ammonium and silicate) was determined in samples preserved by freezing (−20 °C) and then analyzed colorimetrically on a segmented flow system Braun-Luebbe AAII (Grasshoff et al. 1983). Samples for the determination of chlorophyll were collected in dark bottles of 150 ml, stored in a cool, dark place until arrival at the laboratory, where they were filtered through glass fiber filters (GF/F 25 mm in diameter) under vacuum.

Chlorophylls a, b and c were extracted in cold (−20 °C) acetone 90% and quantified on a Perkin Elmer LB-50s spectrofluorimeter using the procedure of Neveux and Panouse (1987). In this study we only consider chlorophyll a values. Particulate organic carbon and nitrogen concentrations (POC, PON) were determined using an elemental analyzer (Carlo Erba CHNSO 1108) on 0.5 to 1 L subsamples vacuum filtered through glass fiber filters (GF/F). Total organic carbon (TOC) was determined in subsamples fixed with 1 mL of H_3_PO_4_ (25% v/v) until a pH <2 to remove inorganic carbon. In the laboratory, samples were analyzed by high-temperature catalytic combustion on a Shimadzu 5000A analyzer (Doval et al. 2016). Dissolved organic carbon concentrations (DOC) were estimated by the difference TOC-POC.

The concentration of two types of DOC (humic acids and amino acids) were estimated from direct measurements of induced fluorescence on a spectrofluorimeter Perkin Elmer LS50 B. Fluorescence values were converted into concentrations in ppb (µg L^−1^) equivalents of quinine sulfate (humic acids) or tryptophan (amino acids) using calibration lines (Nieto-Cid et al. 2005). The samples for these determinations were collected in 15-mL Teflon-caped glass tubes and stored in a cool place until measurement (less than 6 h of collection). In this study the concentrations equivalent to the fluorescence maxima corresponding to generic humic acids (HG, excitation: 250 nm, emission: 435 nm) and tryptophan (TRP, 280, 350 nm) were determined following Nieto-Cid et al. (2005). Humic acids were used as descriptors of dissolved organic matter of low biological degradation (recalcitrant) while TRP was used as an indicator of easily degradable organic matter (Nieto-Cid et al. 2005).

Raw data of physical and chemical variables can be accessed through the PANGAEA repository (https://issues.pangaea.de/browse/PDI-13428 submitted on 04/11/2016).

### Phytoplankton determinations

Taxonomic characterization of phytoplankton was made in samples from three stations representative of the bay (St. 1), estuary (St. 5) and the river–reservoir (St. 10) collected during each of the 10 sampling events. Previous studies in the area showed that the monthly sampling in the selected zones provided information representative of the main states of the phytoplankton community during the year (Casas et al. 1999; Varela et al. 2001). Water subsamples (50 mL) were preserved with Lugol’s solution and kept in darkness until phytoplankton identification and counting using the Utermöhl’s technique (Casas et al. 1999; Varela et al. 2001). Depending on phytoplankton concentration, 10–25 mL of sample were allowed to settle in the Utermöhl chamber for up to 24 h. Observation of samples was carried out using a Nikon Eclipse TE3000 inverted microscope with Nomarsky interference contrast system. Magnification powers of 100×, 200× and 400× were used, according to the size of organisms. The entire slide was examined at 100× to account for large species while only transects or smaller areas were examined at higher magnification. At least 250 cells were counted for each sample. Only well preserved cells were counted, excluding damaged or dead cells (e.g., diatom frustules without visible organic content) that were particularly abundant in the estuarine station. Taxonomical identification was carried out at the lowest (species) level where possible. Species nomenclature was validated according to the World Register of Marine Species (http://www.marinespecies.org).

Values of phytoplankton abundance are available at the PANGAEA repository (https://issues.pangaea.de/browse/PDI-13428 submitted on 04/11/2016).

### Meteorological and hydrological drivers

Daily irradiance and rainfall data were provided by the observatory of the Agencia Española de Meteorología (AEMET) in A Coruña (http://www.aemet.es/). The upwelling intensity was estimated by calculating the Ekman transport from surface winds as an upwelling index (km^3^ s^−1^ km^−1^) computed by the Instituto Español de Oceanografía (http://www.indicedeafloramiento.ieo.es/) in a cell of 1° × 1° centered at 44°N, 9°W, using data from atmospheric pressure at sea level derived from the WXMAP model (González-Nuevo et al. 2014). Positive values of this index indicate net upwelling periods when surface water is transported offshore while negative values indicate an accumulation of surface water against the coast (downwelling). River flow was estimated from the discharge values of the Cecebre reservoir (m^3^ s^−1^) provided by the regional water authority (http://augas.cmati.xunta.es/). Daily discharge values were decreased by 1 m^3^ s^−1^ to account for the average water flow diverged from the river for the urban supply to the city of A Coruña. The water inputs to the Mero river downstream of the reservoir were considered negligible (Gómez-Gesteira et al. 1999). In this study, values of precipitation, upwelling and river discharge were accumulated during 15 days prior to each sampling date. These values represent the accumulated effect of the main meteorological and hydrological factors on phytoplankton dynamics, as shown by other studies in the Galician upwelling (e.g., Nogueira et al. 1997).

### Statistical analysis

Composition and connectedness of phytoplankton assemblages were studied using several diversity indices. At local scale (i.e., for each combination of station and sampling date) species richness (*S*, number of lowest level taxa), Shannon index (bits indiv^−1^) and equitability (evenness with which individuals are divided among the taxa present) indices were computed:

$${\text{Shannon index}} = - \mathop \sum \limits_i \frac{{{n_i}}}{n}\ln \frac{{{n_i}}}{n},$$

where *n*_*i*_ and *n* are the abundance of taxon *i* and total abundance, respectively.

$${\text{Equitability}} = \frac{{{\text{Shannon }}\,\,{\text{index}}}}{{\ln (S)}}$$

Equitability assumes a value between 0 and 1, with 1 being complete evenness (Magurran 2004). For combinations of station and sampling dates we examined two measures of zonal diversity: the number of shared species (co-occurring low-level taxa between two or more stations or sampling dates), and β-diversity (difference between neighboring assemblages) using the index of Harrison et al. (1992):

$$\beta {\text{ - diversity}} = \frac{{\frac{{{S_{\text{p}}}}}{{\bar \alpha }} - 1}}{{N - 1}},$$

where *S*_p_ is the total taxon richness of the pooled *N* set of samples compared and $\bar \alpha$ is their average number of taxa. This index is based on the relative number of taxa and measured the proportional change in richness, reaching maximum values when the percentage of species shared in common between neighboring assemblages is small and the percentages gained and lost are similar (Koleff et al. 2003).

Differences in Shannon index between stations were studied with a modified version of the *t* test (Hutcheson 1970). This test and all diversity indices were computed using the PAST package v 3.0 (Hammer 2015).

Ordination of phytoplankton assemblages was made using multidimensional scaling (MDS) on a Bray-Curtis similarity matrix constructed from log-transformed abundance data after excluding the categories without a clear taxonomic allocation (e.g., microflagellates). Related samples were grouped by hierarchical cluster analysis by applying the group-average method to the similarity matrix. Species characteristic of each group were identified with the SIMPER procedure (Clarke and Warwick 2001). Selection of environmental variables for comparison with phytoplankton data was made after correlation analysis to exclude highly correlated or redundant variables (e.g., ammonium and phosphate, Table 1S in the Supplement). The relationships between normalized environmental variables and phytoplankton taxa for each station were analyzed by the BEST procedure (Clarke and Warwick 2001), based on a weighted Spearman correlation between environmental and phytoplankton abundance similarity matrices. Partition of spatial (station) and temporal (sampling date) variance components of both the environmental and phytoplankton variables was examined with PERMANOVA+ tests on the corresponding similarity matrices (Anderson et al. 2008). All similarity-related analysis were made using PRIMER V 6 (Clarke and Gorley 2006) and PERMANOVA+ (Anderson et al. 2008).

## Results

### Meteorological variability

**Fig. 2:**
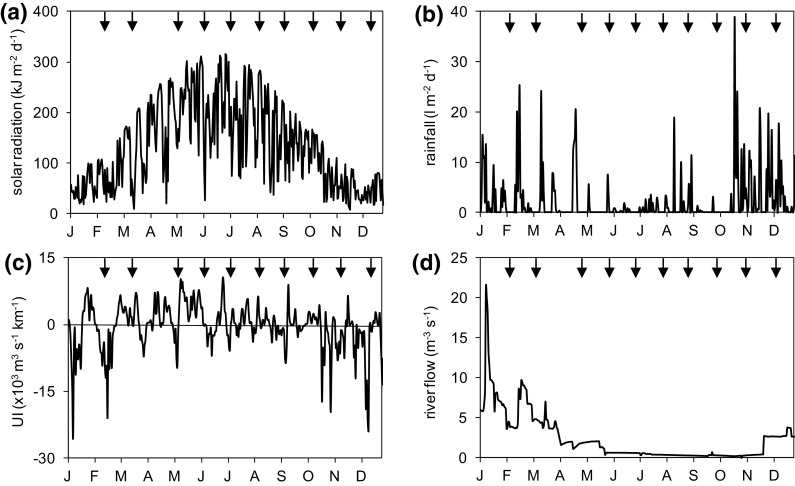
Daily variation of solar radiation (**a**, kJ m^−2^ day^−1^), rainfall (**b**, l m^−2^ day^−1^), upwelling index (**c**, UI, ×10^3^ m^3^ s^−1^ km^−1^) and river flow (**d**, m^3^ s^−1^) in the study area during 2011. The sampling dates for phytoplankton and water variables are indicated by *downward arrows* in each *panel*

The study area is characterized by a seasonal cycle with high values of solar irradiance and upwelling index, and low rainfall and river flow during spring and summer (March to September, Fig. 2). In 2011, there was a relatively long rainfall period from mid October–December in addition to the episodic rains recorded in previous winter and spring (January–May). Several upwelling events occurred along the year but more persistently during spring and summer, while downwelling prevailed in winter and also in autumn. The river flow showed high values during the winter–spring period but was between 0.5 and 3 m^3^ s^−1^ for most of the summer and autumn (Fig. 2c). River flow was uncorrelated with rainfall when data from the same calendar day were compared but showed a positive correlation at lags of up to 7 days, with a maximum correlation value with the rainfall recorded 5 days earlier (*r* = 0.212, *N* = 365, *P* < 0.05). This latter correlation, along with the low flow measured during spring and summer points out to the major role of the reservoir in regulating the freshwater flow to the estuary, while attending the demands for urban freshwater and flood control during periods of heavy rain. When accumulated in periods of 15 days, values of upwelling index were also negatively correlated with rainfall and positively with irradiance (Table 1S in the Supplement). Either precipitation, irradiance or upwelling index were significantly correlated when accumulated in periods of 7, 15 or 30 days (values not shown); therefore, values accumulated for 15-day periods prior to each sampling date were used in subsequent analysis.

### Variability in physical and chemical properties of water

**Fig. 3:**
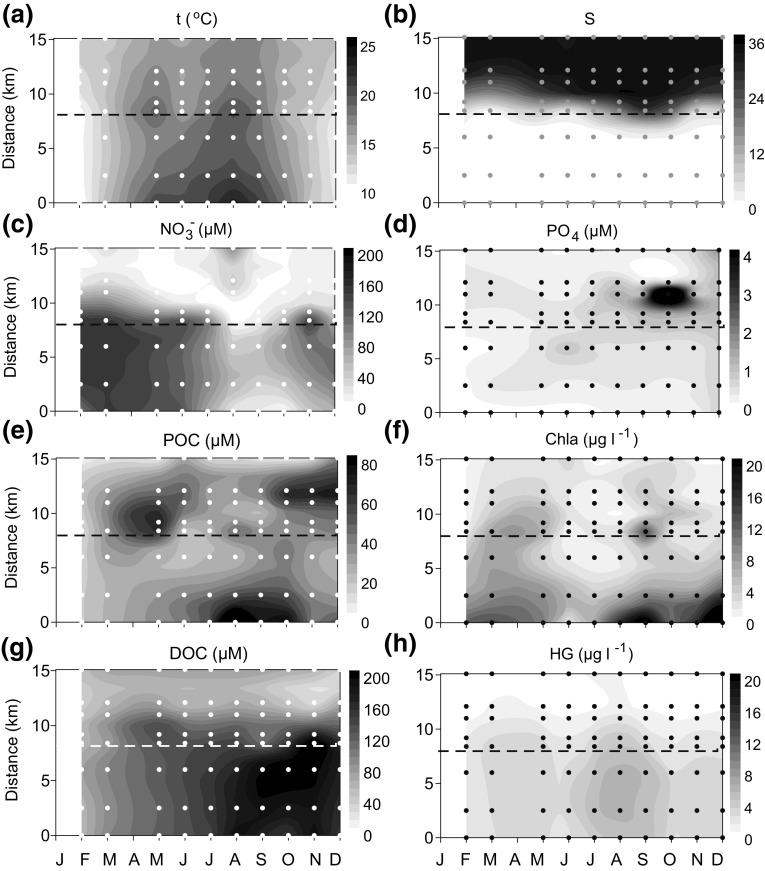
Distribution of water temperature (**a**, *t*, °C), salinity (**b**, S), nitrate (**c**, NO_3_, µM), phosphate (**d**, PO_4_, µM), particulate organic carbon (**e**, POC, µM), chlorophyll *a* (**f**, Chla, µg L^−1^), dissolved organic carbon (**g**, DOC, µM) and humic acids (**h**, HG, µg quinine sulfate-eq. L^−1^) along the salinity gradient during 2011. Distance between stations (km) is referred to the reservoir (St. 10). The *dashed line* indicates the location of St. 5

The thermal cycle of progressive warming of surface water during spring and summer, and cooling during autumn and winter was more pronounced in the river and reservoir compared to the bay and estuary (Fig. 3a). The seasonal variability in salinity was reduced when compared with the large spatial variability, with a marked saline front delineating the influence of the saline waters in the estuary near the location of St. 5 (Fig. 3b). The range of salinity values observed was 3.0, 28.3 and 0.0 at stations 1 (bay), 5 (estuary) and 10 (reservoir), respectively. These values are considerably smaller than the range of salinity observed across the 15 km separating the bay station from the reservoir at all sampling times (salinity range >35). Similarly, all water variables mainly showed spatial gradients, while temporal (i.e., seasonal) variability was comparatively smaller (Table 2S in the Supplement). For instance, nitrate had higher concentrations in freshwater than in marine water (Fig. 3c), and phosphate displayed maximal values in the estuary (Fig. 3d). However, relative increases in nitrate concentrations in the bay and decreases in the estuary and river waters during summer must be noticed. Phosphate concentrations were more variable near the saline front in the estuary. Silicate and ammonium concentrations (not shown) displayed similar variability to either nitrate or phosphate, respectively, as indicated by their correlations (Table 2S in the Supplement).

Maximum values of particulate organic matter concentrations were found in the estuary in spring, in the river and reservoir in late summer and in autumn in the bay (Fig. 3e). In general, POC was significantly correlated with chlorophyll a (Table 1S in the Supplement), which always showed the highest values in the reservoir (Fig. 3f). However, concentrations exceeding 5 µg L^−1^ indicated blooms during spring and late summer in the estuary and in the bay. In turn, dissolved organic matter was always higher in freshwater, with concentrations increasing during spring and summer, reaching maximum values during autumn (Fig. 3g, h).

### Phytoplankton communities

A total of 130 phytoplankton taxa were identified (118 at least at genus level), including 63 diatoms (Bacillariophyceae), 32 dinoflagellates (Dinophyceae), 25 Chlorophyceae and other groups with less than 10 taxa each (Table 1, 3S). Considering the entire annual cycle, the number of taxa decreased progressively from the bay (St. 1) to the reservoir (St. 10) while the Shannon index reached minimum values in the estuary (St. 5), which also showed the highest abundance (Table 1). There was an even distribution of abundance among taxa in the bay, moderately even in the reservoir but highly uneven in the estuary, as indicated by the values of equitability. The differences in Shannon index were significant for all pairs of stations (Hutcheson *t* test, *t* St. 1–5 = 470.73, *t* St. 5–10 = −1810.8, *t* St. 1–10 = 386.93, *P* < 0.001 in all cases). Unique taxa (i.e., those found only at one of the sampling stations) accounted for more than half of all taxa recorded in the bay and the reservoir, but only 35% of those found in the estuary (Table 1).

**Table 1:** Number of taxa of the lowest level (species whenever possible) of phytoplankton groups, accumulated abundance (×10^7^ cells mL^−1^), H (Shannon index, bits indiv^−1^) and equitability (bits indiv^−1^ taxa^−1^) observed at each station

	Group	St. 1 (bay)	St. 5 (estuary)	St. 10 (reservoir)
Taxa (number)	Charophyta	0	0	2
	Chlorophyceae	0	4	14
	Cryptophyceae	1	3	3
	Chrysophyceae	0	0	1
	Synurophyceae	0	0	1
	Cyanophyceae	0	1	5
	Bacillariophyceae (Diatoms)	47	31	12
	Dictyochophyceae	1	0	0
	Dinophyceae (Dinoflagellates)	28	13	3
	Euglenoidea	1	2	0
	Incertae sedis (*Solenicola setigera*)	1	0	0
	Prymnesiophyceae	2	0	0
	Xanthophyceae	0	0	1
	Total	81	54	41
	Unique taxa	52	19	23
Abundance (×10^7^ cells mL^−1^)		0.01	6.87	3.58
H (bits indiv.^−1^)		2.22	0.03	0.42
Equitability (bits indiv.^−1^ taxa^−1^)		0.51	0.01	0.11

Diversity indices were computed after integrating all samples by station. The total number of taxa and the number of taxa found exclusively in each station (unique taxa) are also given

**Fig. 4:**
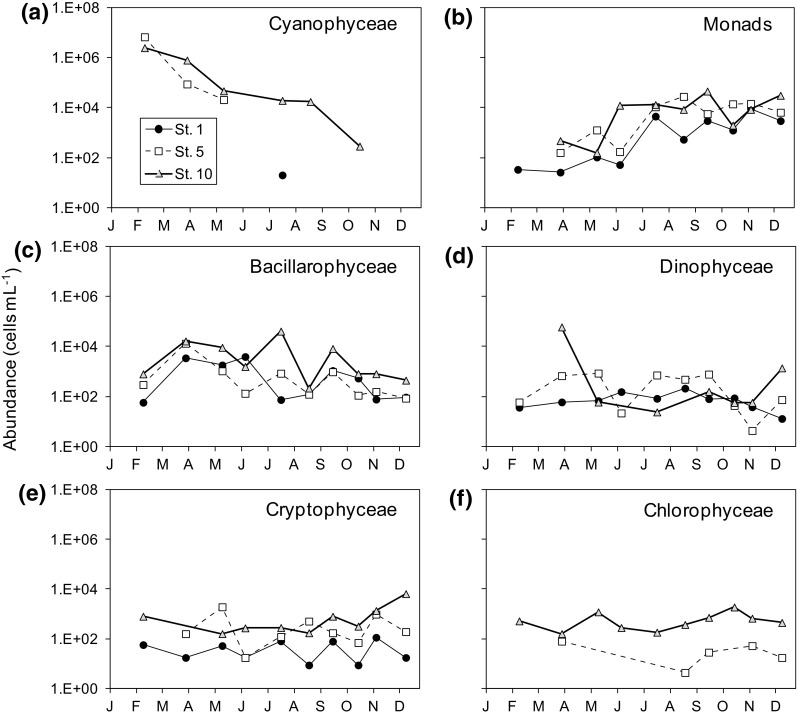
Seasonal variations in abundance of the main phytoplankton groups (cells mL^−1^) in the sampled stations during 2011. **a** Cyanophyceae, **b** Monads (2–8 µm), **c** Bacillarophyceae, **d** Dinophyceae, **e** Cryptophyceae, **f** Chlorophyceae. Note the logarithmic scale of abundance in all *panels*

Most of the variations in abundance were due to cyanobacteria (Cyanophyceae), almost permanent in the reservoir but also present in the estuary and even reaching the bay (Fig. 4a). Cyanophyceae, mainly *Chroococcus* spp. (Table 3S in the Supplement), reached maximum abundance in February and decreased during spring and summer. The second group in abundance was composed by small (2–8 µm) flagellate monads which increased in abundance from February to December in all zones (Fig. 2b). This group was not employed in further analysis because it was not possible to separate the autotrophic and heterotrophic organisms with the counting technique employed. Apart from these groups, the phytoplankton communities were dominated by Bacillariophyceae (Fig. 4c), Dinophyceae (Fig. 4d) and Cryptophyceae (Fig. 4e) in all zones and Chlorophyceae in the stations under the influence of freshwater (Fig. 4f). The dominant taxa were the diatoms *Chaetoceros socialis, C. affinis* and *Pseudo-nitzschia pungens* at St. 1 (spring), and the cyanobacteria *Chroococcus* spp. (spring) and the Euglenoidea *Eutreptia* sp. (October) at St. 5. In the reservoir, there were also dominance peaks of *Chroococcus* spp. in spring that were later replaced by other cyanobacteria, *Anabaena spiralis* (August), the freshwater diatom *Fragilaria crotonensis* (June and July) and the Synurophyceae *Synura uvella* (September) at St. 10 (Table 2S in the Supplement).

**Fig. 5:**
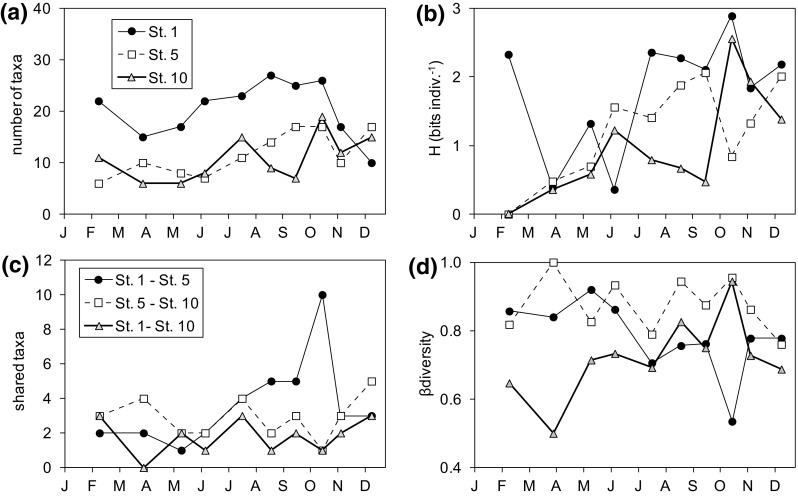
Seasonal variations in diversity in the sampled stations during 2011. **a** Number of taxa, **b** H (Shannon index, bits indiv.^−1^), **c** number of shared taxa between pairs of stations, **d** β-diversity (Harrison et al. 1992). *Symbols* for *panels* **a** and **b** are different from those in **c** and **d**

The number of taxa and Shannon index values increased in general from spring to late summer at all stations, with the highest values almost always in the bay and the lowest in the reservoir (Fig. 5a, b).There were taxa present in several zones (Fig. 5c); only a few taxa were shared between the estuary and the reservoir at any single sampling time, while the bay and the estuary showed an increase from spring to autumn in the number of taxa present in both zones. Four of the taxa shared were high-level taxa, not resolved at the species level, as Cryptophyceae or unidentified dinoflagellates and diatoms, but in all cases, there were characteristic species that were found in several stations (Table 3S). For instance, *F. crotonensis* was identified not only in the reservoir and estuary but also in the bay. The highest number of shared species occurred when comparing the bay and the estuary stations. Conversely, β-diversity showed high values for the assemblages of St. 5 and St. 10 through the year and also for those of St. 1 and St. 5 in spring (Fig. 5d) when the number of shared species was relatively low (Fig. 5c). In the later, the decrease of β-diversity in summer and autumn was accompanied by a sharp increase in the number of shared taxa. However, this correspondence was lower in the case of the assemblages of end-member stations (St. 1 and St. 10) showing relatively low β-diversity values and low numbers of shared taxa. Considering the entire sampling period, 8 taxa occurred in all zones and the estuary shared 24 taxa with the bay and 19 with the reservoir, while the corresponding values of β-diversity were 0.61 (all zones combined), 0.64 (St. 1 vs. St. 5) and 0.62 (St. 5 and St. 10).

**Fig. 6:**
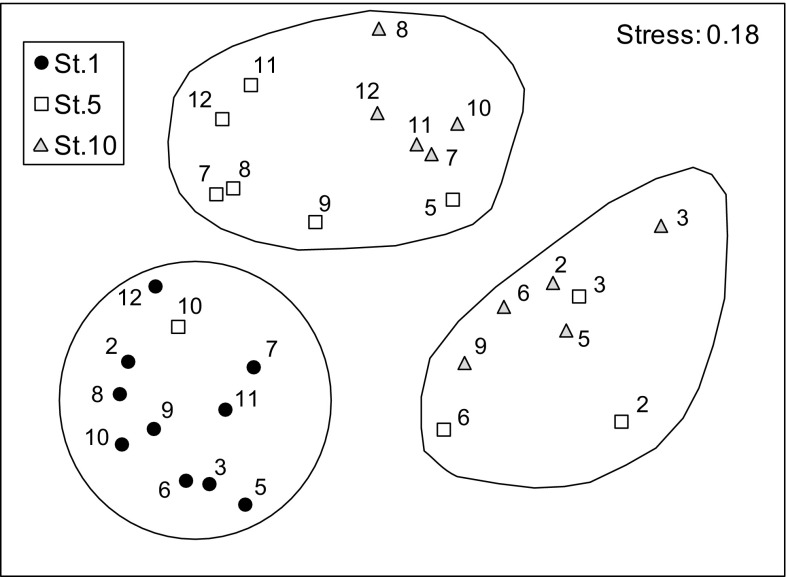
MDS plot of phytoplankton samples (*numbers* indicate the sampling month and colors and *shapes* represent the different stations) grouped by 20% similarity in species composition (*dashed line*) according to the group-average clustering (see Fig. 1S in the Supplement)

The taxonomic composition defined the characteristics of each sampling zone throughout the year, as shown both by the MDS (Fig. 6) and cluster analysis (Fig. 1S in the Supplement). As observed for environmental variables, the composition of phytoplankton communities varied mainly with the spatial component (i.e., station) while the temporal variability was comparatively smaller (Table 2S in the Supplement). The samples from the bay (St. 1) were at all times clearly separated from the other stations (Fig. 6) and their similarity was mainly due to medium-sized Cryptophyceae, the diatom *Nitzschia longissima* and small dinoflagellates (Table 2). Samples from the estuary (St. 5) and the reservoir (St. 10) were also separated but in this case, there were more similarities in the composition of the communities between stations in some periods of the year. For instance, half of the samples from the reservoir clustered with either summer and autumn samples or winter and spring samples of the estuary at the 20% similarity level (Fig. 6). The main contributors to the similarity of St. 5 were Cryptophyceae, small diatoms, *N. longissima*, and *F. crotonensis*, which was also the main contributor to the similarity of St. 10 along with the Chlorophyceae *Ankistrodesmus falcatus*, Cryptophyceae and small dinoflagellates (Table 2). It must be noted that the contribution of most taxa to similarity within stations was small (<1%), while the main contributors were none of those identified above as bloom producers.

**Table 2:** Mean and SE abundance (cells mL^−1^) of the main phytoplankton species contributing to the similarity (Sim %.) within stations determined with the procedure SIMPER (Clarke and Warwick 2001)

Species	Group	St. 1 (bay)			St. 5 (estuary)			St. 10 (reservoir)		
		Mean	SE	Sim %	Mean	SE	Sim %	Mean	SE	Sim %
*Ankistrodesmus falcatus*	Chlorophyceae	0.0	0.0	*	42.2	37.8	*	140.4	22.8	9.13
Cryptophyceae >20 µm	Cryptophyceae	0.0	0.0	*	446.0	207.9	14.32	456.1	225.0	5.81
Cryptophyceae 10–20 µm	Cryptophyceae	45.6	11.7	20.49	82.7	41.6	*	508.2	170.0	5.43
Cryptophyceae <10 µm	Cryptophyceae	0.0	0.0	*	203.6	92.4	11.10	1264.8	992.2	5.58
Diatoms pennate < 20 µm	Bacillariophyceae	14.9	6.9	*	27.6	4.0	8.92	0.0	0.0	*
Diatoms pennate >20 µm	Bacillariophyceae	4.9	2.9	*	60.5	30.9	10.43	44.5	15.5	*
Dinoflagellates <20 µm	Dinophyceae	38.7	9.7	19.71	152.4	64.5	*	64.4	25.2	4.03
Dinoflagellates >20 µm	Dinophyceae	7.0	1.7	7.31	155.5	122.1	6.08	31.9	15.5	*
*Fragilaria crotonensis*	Bacillariophyceae	8.0	0.0	*	3527.0	3068.1	5.31	6372.8	3760.1	36.77
*Nitzschia longissima*	Bacillariophyceae	5.8	1.9	8.09	10.8	10.6	3.56	0.0	0.0	*

*Contributions to similarity <1%

### Environmental effects on the phytoplankton communities

Salinity, nitrate, and dissolved organic carbon concentration were the main variables correlated with the composition of phytoplankton communities when all zones were considered (Table 3). In the bay, where variations in salinity were relatively low, the main variables contributing to the correlation between environmental and taxonomic data were the concentration of phosphate and dissolved organic matter. In the estuary, salinity, phosphate, and concentrations of humic acids and tryptophan-like substances contributed to a relatively high correlation, compared to that found in other stations. In turn, nitrate, phosphate and organic matter components were the main environmental variables correlated with phytoplankton composition in the reservoir.

**Table 3:** Best correlations (weighted Spearman coefficient) between environmental and species abundance similarity matrices for total and individual stations computed using euclidean distance (procedure BEST in PRIMER, Clarke and Gorley 2006)

Analysis	Spearman	Variables			
All stations	0.627	S	NO3	DOC	
St. 1 (bay)	0.423	PO4	HG	TRP	DOC
St. 5 (estuary)	0.821	S	PO4	HG	TRP
St. 10 (reservoir)	0.579	NO3	PO4	HG	TRP

The main environmental variables contributing to the correlation are indicated for the whole dataset and for each subset

*S* salinity, *NO3* nitrate, *PO4* phosphate, *DOC* dissolved organic carbon, *HG* generic humic acids, *TRP* tryptophan-like amino acids

**Fig. 7:**
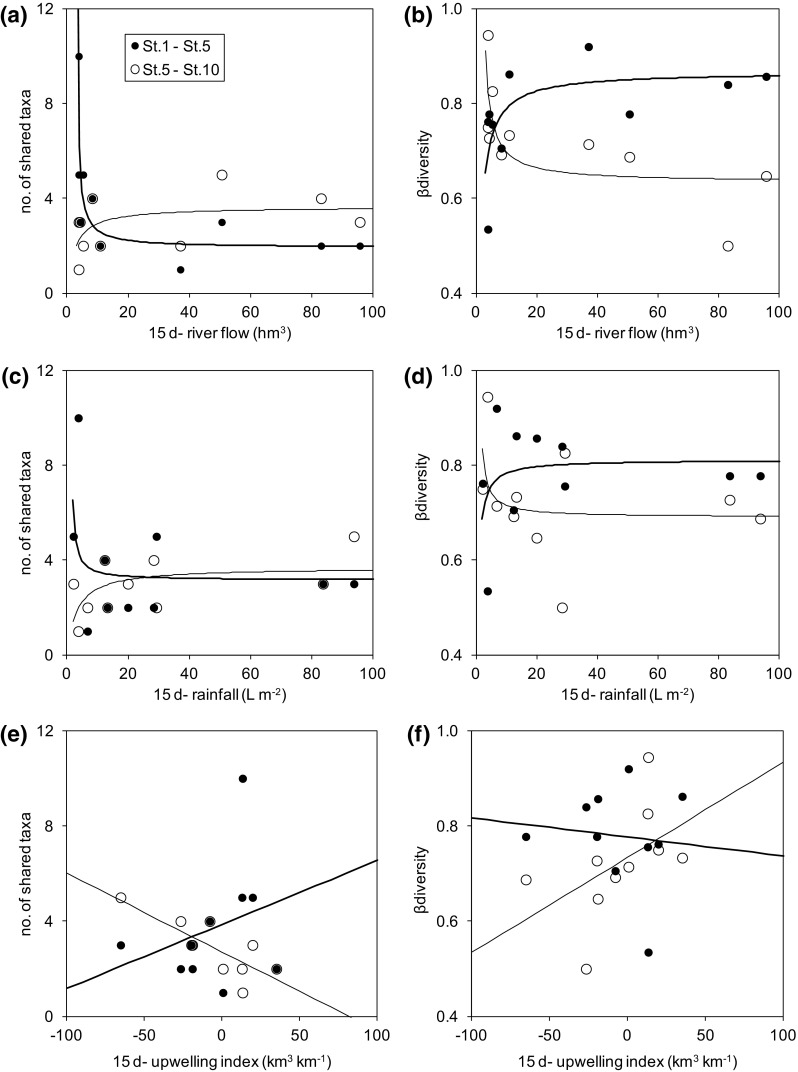
Variation in the number of taxa shared and β-diversity of St. 5 and St. 1 or St. 10 in relation to river flow (**a**, hm^3^), rainfall (**b**, L m^−2^) or upwelling index (**c**, km^3^ km^−1^) accumulated for 15 days prior to sampling. Significant (*p* < 0.05) regression lines and equations are displayed for descriptive purposes (fitting parameters and statistics are provided in Table 4S and Fig. 2S in the Supplement)

In general, the meteorological variables showed a low correlation with phytoplankton taxa, but there was asymmetric covariation of zonal diversity indices with the meteorology (Fig. 7; Table 4S; Fig. 2S in the Supplement). Rainfall and river flow were related with both the number of shared taxa and β-diversity by a saturation-type function, but the pattern was different for the combination of the bay and estuarine stations or the estuarine and the reservoir stations. In the former case, there was a rapid decrease of the number of shared taxa (and conversely an increase in β-diversity) with the increase in river flow (Fig. 7a, b). Apparently, the effects on zonal diversity depend on a critical value of the flow (ca. 20 hm^3^ per 15 days). River flow values larger than this critical value had little effect on zonal diversity while there were large changes at lower flow values. For instance, when the flow was lower than the critical value there were more taxa shared between the bay and the estuary than during periods of high flow (Mann–Whitney test, *p* < 0.05, *n* = 10). Conversely, there was a slight increase in the number of shared taxa (and a decrease in β-diversity) between the estuary and the reservoir when the river flow exceeded the critical value. Similar patterns could be applied to the accumulated rainfall, but with lower confidence than for the river flow (Fig. 7c, d; Table 4S). In contrast, upwelling did not show the described saturating response and its covariation with zonal diversity indices was less clear (Fig. 7e, f; Table 4S). The only significant effect of upwelling was a linear and negative effect on the number of taxa shared between the estuary and the reservoir (Fig. 7e).

**Fig. 8:**
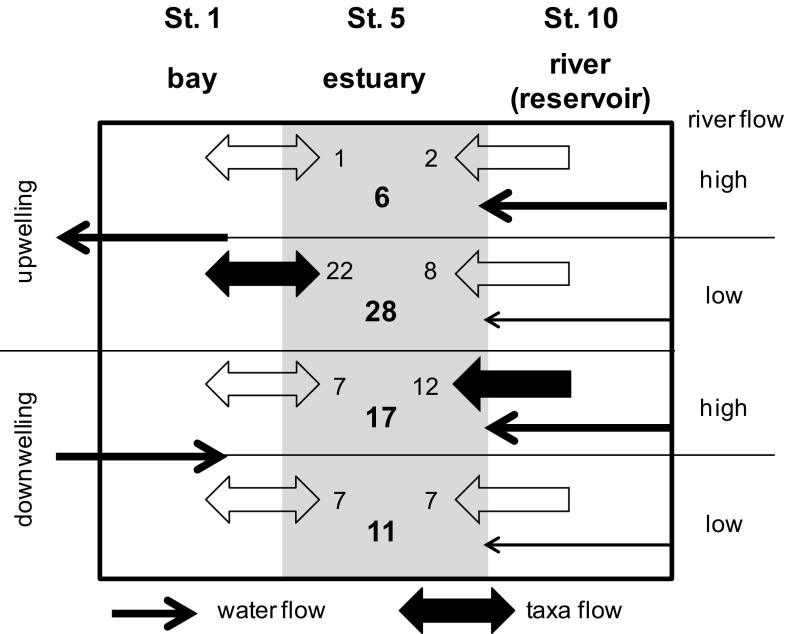
Summary of the influence of upwelling and river flow on the number of taxa found in the estuary (St. 5). *Arrows* indicate the water and taxa flows during periods of positive (upwelling) or negative (downwelling) UI values, and high (>20 hm^3^) or low (<20 hm^3^) river flow accumulated in the 15 days prior to phytoplankton sampling (as in Fig. 7). *Thickness* and *filling of arrows* indicate the dominant flows in each case. *Numbers* indicate the number of taxa shared with the bay (St. 1) or the reservoir (St. 10) and the number of unique estuarine taxa (*bold numbers*) for each period

The combination of river flow and upwelling conditions thus affected the number of phytoplankton taxa found in nearby zones (PERMANOVA+ test, Table 5S in the Supplement). As summarized in Fig. 8, the number of taxa shared between the bay and the estuary peaked in periods of upwelling and low river flow (<20 hm^3^ in 15 days) when also the number of estuarine taxa reached a maximum. In turn, the maximum number of taxa shared between the estuary and the reservoir was found in downwelling conditions and during periods of high river flow (≥20 hm^3^ in 15 days). For all combinations of upwelling and river flow, the number of taxa found only in the estuary was higher than the number of taxa shared with the other zones.

## Discussion

### Unique vs. imported species in the estuary

The results of this study align with the current paradigm of salinity as a primary environmental driver of estuarine communities taking into account also the interactions with hydromorphology (Elliot and Whitfield 2011). Tolerance to salinity variations rather than tolerance to a specific salinity is thus the principal environmental factor regulating the distribution of estuarine organisms, including phytoplankton. Taxonomic composition and phylogenic relatedness of estuarine phytoplankton communities are strongly correlated to salinity gradients (Olli et al. 2015). Because of the large range of salinity variation, only a subset of species is well adapted to the salinity range experienced at a given location (e.g., Balzano et al. 2011). As found in the present study, minimum values of local diversity (Shannon index and equitability but not in species richness) occurred in the estuarine zone characterized by the highest salinity gradient. Other studies in estuaries, however, found no local minima of phytoplankton diversity near the salinity front but reported increases in zonal diversity that were attributed to the contribution of allochtonous taxa from nearby locations (Muylaert et al. 2009). In our study, some of the taxa from the sea and the river imported to the estuary may eventually did not survive because of the salinity changes but still form a pool of taxa increasing the species richness. However, these rare taxa will have low impact on Shannon index values because this index weights taxa proportionally to their abundance. While our results also indicate a large number of taxa shared between the estuary and the other zones, almost a third of the total number were unique to the estuary, which can be also considered an ecotone because of the abrupt change in salinity conditions. Previous studies in the Galician rias have stressed the major role of upwelling–downwelling cycles on the composition of phytoplankton assemblages by means of changes in water column stratification and its influence on the nutrient availability (e.g., Figueiras and Pazos 1991; Nogueira et al. 2000), but most studies did not include samples in the zone of maximum salinity gradient nor in the contributing rivers. Only a few studies reported the presence of freshwater species, generally associated to runoff in periods of intense rainfall (Varela et al. 2001, 2004, 2005). The inclusion of communities from the end-member zones of the salinity gradient in this study allows for a first analysis of the importance of the connectivity of these potential sources of taxa in determining the composition of phytoplankton assemblages in the estuary.

The taxa found in this study were already described in previous studies in marine (e.g., Casas et al. 1999; Nogueira et al. 2000; Varela et al. 2001, 2005; Varela and Prego 2003) and freshwater habitats (e.g., Vasconcelos and Cerqueira 2001; Negro et al. 2000) in this region. The most characteristic taxa in all zones were diatoms, along with increasing importance of Cryptophyceae and Chlorophyceae in the zones with highest freshwater influence, as reported for other estuaries (Cloern and Dufford 2005; Ferreira et al. 2005; Muylaert et al. 2009; Carstensen et al. 2015; Sin and Jeong 2015). Diatom dominance is expected when high nutrient supply and turbulence conditions prevail but also in areas of siliceous rocks as in Galicia, where they are the main bloom-forming species (Varela and Prego 2003; Varela et al. 2004, 2005). More interesting is the importance of Cryptophyceae as characterizing taxa for the different communities. Even when the species identification by morphological details is not generally achieved, Cryptophyceae have been used as indicators of major changes in estuarine communities (Seoane et al. 2012; Brito et al. 2014; Šupraha et al. 2014; Sin and Jeong 2015) and molecular studies further illustrate the relevance of this taxon for the analysis of changes in phytoplankton diversity (Bazin et al. 2014). The increasing presence of Cryptophyceae in estuaries and bays has been related to the warming (Brito et al. 2014) and eutrophication of waters mainly due to increasing loads of phosphate (Šupraha et al. 2014; Sin and Jeong 2015) and nitrogen (Brito et al. 2014). In addition, the presence of high numbers of Cyanophyceae in the estuary during periods of high river flow was reported here for the first time for a Galician ria, while it seems a common feature of estuaries receiving much large river inputs (Galvão et al. 2008). The abundance of taxa from Cryptophyceae and Cyanophyceae found in our study challenges the expected dominance of diatoms in the estuary and bay areas and suggests a potential eutrophication of these areas due to river inputs.

Salinity was thus the major environmental factor affecting phytoplankton community distribution by the selection of species characteristic of each salinity domain. Nitrate was the main nutrient correlated with changes in phytoplankton assemblages through the salinity gradient, as expected not only by the influence of the upwelling in the marine domain (Figueiras and Pazos 1991; Varela et al. 2001, 2004, 2005; Varela and Prego 2003) but also in the reservoir. Cyanobacterial blooms during summer and autumn are a common feature of most reservoirs in the region and can be related, at least in part to nitrogen and phosphorous availability (Galvão et al. 2008). In other estuaries, cyanobacteria (including picocyanobacteria) reach peak biomass levels during summertime, when the temperature is maximal and there is an increase in inorganic phosphorus released from the sediments (Gaulke et al. 2010). However, we found unusual patterns of cyanobacterial abundance in the reservoir, with maximal abundances in winter which affected the estuarine zone. These winter blooms could be induced by the accumulation of phosphate in the previous autumn, as suggested by the increase in phosphate observed during the study in the reservoir and the estuary. The co-occurrence of minimum phosphate concentrations and maximum abundance of cyanobacteria also supports this hypothesis. In addition, phosphate concentration was correlated with phytoplankton assemblages in the estuary (Table 3), where local maximum concentrations in late summer and autumn may be related to point sources (González 1975), but otherwise (as silicate), its correlation was not particularly high in the other zones. Because of the high concentrations of nutrients found in the entire salinity gradient (up to 160 µM for nitrate or silicate and 10 µM for phosphate) compared with those observed in typical coastal waters (e.g., Varela and Prego 2003) nutrient limitation does not appear a direct factor influencing phytoplankton composition in our study area, or at least with less influence than hydrological fluxes. In contrast, other studies attributed a major role to the alteration of nutrient ratios as one of the main causes of change in the taxonomic composition of phytoplankton assemblages in estuaries influenced by reservoirs (e.g., Galvão et al. 2008) and in rias influenced by upwelling (Figueiras and Pazos 1991). Seasonal accumulations of organic matter have been described in the region as the result of biogeochemical processing of phytoplankton blooms occurring in spring and summer, both in the rias and coastal waters (Nieto-Cid et al. 2005; Bode et al. 2005; Doval et al. 2016) and in reservoirs (Gago et al. 2005).

### Hydrological effects on diversity patterns

Notwithstanding the existence of locally adapted species, water fluxes strongly influenced the connectedness of phytoplankton assemblages in the estuary. The different measurements of diversity provide complementary descriptors of the communities. While the number of taxa shared between zones is one of the most intuitive and explored measures (Koleff et al. 2003) it only records the continuity in the taxonomic composition but does not take into account the turnover of species, i.e., the gains and losses between zones. In contrast, the β-diversity index selected in this study (Harrison et al. 1992) provides a measure of turnover because it measures the proportional change in richness rather than absolute changes in the species pool (total richness). Taxonomic turnover (and β-diversity values) between two zones is high when there is a low fraction of taxa shared and the relative gains and losses are similar (Koleff et al. 2003). This is observed in this study as the negative correlation between shared taxa and β-diversity values. Other measures of zonal diversity relied on the rate of change of taxa with the distance between zones, as shown by Muylaert et al. (2009) in estuarine phytoplankton. In this case, maximum values of zonal diversity were found in zones with frequent inputs of species from other domains (thus focusing on species gains). Our results also agree with the prediction of a decrease in zonal diversity (β-diversity) with increasing connectivity between zones differing in taxonomic composition (Sin and Jeong 2015).

Connectedness of the studied phytoplankton communities was affected by the combination of fluxes driven by upwelling and river dynamics (Fig. 8). This result agrees with the effects of habitat connectivity on estuarine phytoplankton in regions affected by coastal upwelling, but described at larger time and space scales (Cloern and Jassby 2010). In contrast, phytoplankton in other estuaries was only affected by river flow and tides. Tidal mixing can be the main driver of taxa distribution in the water column over imposing its effects on the salinity gradient (Brito et al. 2014) while freshwater inflows greatly modify phytoplankton diversity (Muylaert et al. 2009; Bazin et al. 2014; Brito et al. 2014; Dorado et al. 2015; Sin and Jeong 2015) by altering the residence time of water and species in each zone (Ferreira et al. 2005). Our study points out to a new role of upwelling in determining the composition of phytoplankton assemblages in the Galician rias. Upwelling–downwelling cycles increase connectivity between the estuary and both the bay and the river, but reservoir discharges only increase connectivity with the river. Maximum connectivity will be expected when salinity gradients are maintained in the estuary during periods of downwelling and neap tide. All these hydrological processes displace biological populations and their functions through the whole salinity gradient, transcending the value of individual habitat types, as shown with phytoplankton productivity in estuaries (Lopez et al. 2006). The hydrology drivers facilitate the survival of the estuarine assemblages even when local or regional conditions change, by ensuring a continuous supply of species from source zones (Aiken and Navarrete 2011). However, high water fluxes and mixing do not immediately favor local and zonal adaptation, as shown for estuaries receiving freshwater flushes from reservoirs (Ferreira et al. 2005; Galvão et al. 2008; Sin and Jeong 2015) or rivers (Brito et al. 2014; Dorado et al. 2015), as adaptation is maximized at intermediate levels of connectedness (Leibold and Norberg 2004). In this context, estuaries receiving different hydrological influences, such as the one shown in this study, are model systems to analyze the response of ecosystems to multiple drivers. The connectedness of such systems has major implications for management. For instance, the results of this and previous studies indicate that the regulation of freshwater discharges by reservoirs greatly affects phytoplankton assemblages along the entire salinity gradient and even with delayed effects after the peak in the discharge (Sin and Jeong 2015). In addition, upwelling affects the transport of cells and nutrients but also interacts with local drivers. One example is the variability in the use of anthropogenic versus marine nutrients caused by the confinement of phytoplankton in the estuary by upwelling (Cloern and Jassby 2010). Also local food webs can be affected, as filter-feeders may be able to control the increase of phytoplankton populations despite the availability of nutrients (Lucas et al. 2016). While upwelling and runoff are largely regulated by climatic factors operating at regional scale that are difficult to overcome, the management of local drivers, as freshwater fluxes, must maintain moderate levels of connectivity to maximize phytoplankton diversity at regional scale.

### Challenges for future monitoring

Complete assessment of phytoplankton diversity is a major challenge. The classical morphological identification of species is limited to relatively large organisms (generally >10 µm) because of the limitations of microscopical techniques and the lack of enough external differences in the flagellated forms (e.g., Casas et al. 1999; Varela et al. 2001). Molecular techniques allow much greater taxonomical detail but were rarely implemented in field studies (Bazin et al. 2014). In addition, sample size is a limitation when recording rare species (e.g., Rodriguez-Ramos et al. 2014). While these limitations can be overcome in part by statistical approaches, as the use of rarefaction methods (Magurran 2004), the consideration of species traits in addition to simple records of abundance or biomass will reveal different ecological strategies among major phytoplankton taxa and their response to environmental changes, as shown for cell size (Segura et al. 2013). Monitoring the resilience of estuarine phytoplankton must include all zones connected by the local and regional hydrology, as the intensity of the taxonomic fluxes is largely determined by the fluxes of water.

## Conclusions

The phytoplankton assemblages in the transitional waters system of A Coruña are the result of the seasonally variable influence of marine and freshwater components driven by the relative strength of upwelling and river flow discharges. However, the moderate connectedness of local assemblages allows the persistence of unique taxa at local scales. Consequently, local and zonal diversity patterns vary seasonally and are not simply related to the salinity gradient driven by the river flow, as found in other estuaries. These results suggest that alteration of the hydrologic regime by either influencing freshwater discharges, rainfall or upwelling dynamics would modify connectedness of phytoplankton communities in transitional waters affected by these drivers. The final effect on the species diversity and composition would depend on the resilience of the assemblages, implying the analysis of phytoplankton diversity at increasing spatial and temporal scales.

## Electronic supplementary material

Below is the link to the electronic supplementary material.

Supplementary material 1 (PDF 547 KB)
