# Supplementary material for: The relative effects of upwelling and river flow on the phytoplankton diversity patterns in the ria of A Coruña (NW Spain)
Source: Mar Biol. 2017 Mar 30;164(4):93. doi: 10.1007/s00227-017-3126-9 (PMC5374173; doi:10.1007/s00227-017-3126-9)
Supplement: Supplementary file 1 — Supplementary material 1 (PDF 547 KB) [file 227_2017_3126_MOESM1_ESM.pdf]

Electronic supplementary material

The relative effects of upwelling and river flow on the phytoplankton diversity patterns in the ria of A Coruña (NW Spain)

Antonio Bode*, Manuel Varela, Ricardo Prego, Fernando Rozada, Martin D. Santos

*Corresponding author: antonio.bode@co.ieo.es

Table 1S. Correlation (Pearson's r) between environmental variables (lower semimatrix) and significance values (upper semimatrix) for all sampling stations and dates combined. t: temperature, Sal: salinity, Chla: chlorophyll a, NO_3: nitrate, NO_2: nitrite, PO4: phosphate, NH_4: ammonium, SiO_4: silicate, HG: humic acids, TRP: tryptophan-equivalent fluorescent organic matter, POC: particulate organic carbon, PON: particulate organic nitrogen, DOC: dissolved organic carbon, P_{15}: precipitation accumulated during 15 days prior to sampling, UI_{15}: accumulated Ekman transport during 15 days prior to sampling; R_{15}: accumulated solar radiation during 15 days prior to sampling, F_{15}: accumulated river flow during 15 days prior to sampling. Correlation values with $P<0.05$ where indicated in boldface.

	t	Sal	Chla	NO_3	NO_2	PO_4	NH_4	SiO_4	HG	TRP	POC	PON	DOC	P_{15}	UI_{15}	R_{15}	F_{15}
t	----	0.515	0.793	0.321	0.184	0.249	0.406	0.016	0.009	0.000	0.001	0.004	0.047	0.009	0.000	0.000	0.000
Sal	-0.124	----	0.003	0.000	0.147	0.118	0.328	0.045	0.000	0.032	0.001	0.002	0.000	0.680	0.650	0.830	0.954
Chla	0.050	**-0.522**	----	0.972	0.000	0.638	0.545	0.840	0.034	0.026	0.005	0.001	0.030	0.287	0.232	0.249	0.251
NO_3	-0.188	**-0.613**	0.007	----	0.797	0.353	0.363	0.000	0.132	0.415	0.802	0.571	0.402	0.818	0.509	0.958	0.666
NO_2	-0.249	-0.271	**0.717**	0.049	----	0.118	0.839	0.784	0.563	0.651	0.298	0.224	0.204	0.004	0.003	0.031	0.056
PO_4	-0.217	0.292	-0.090	-0.176	0.292	----	0.000	0.779	0.855	0.912	0.992	0.840	0.817	0.004	0.016	0.011	0.013
NH_4	-0.157	0.185	-0.115	-0.172	0.039	**0.680**	----	0.850	0.840	0.785	0.919	0.849	0.372	0.056	0.391	0.068	0.098
SiO_4	**-0.437**	**-0.369**	-0.038	**0.699**	-0.052	-0.054	-0.036	----	0.310	0.351	0.440	0.383	0.700	0.973	0.236	0.163	0.245
HG	**0.468**	**-0.696**	**0.389**	0.281	0.110	-0.035	-0.038	0.192	----	0.000	0.000	0.000	0.000	0.822	0.802	0.349	0.253
TRP	**0.658**	**-0.393**	**0.405**	-0.155	0.086	-0.021	-0.052	-0.176	**0.798**	----	0.000	0.000	0.000	0.679	0.205	0.122	0.121
POC	**0.565**	**-0.571**	**0.503**	-0.048	0.196	0.002	-0.019	-0.146	**0.653**	**0.758**	----	0.000	0.000	0.817	0.630	0.404	0.613
PON	**0.514**	**-0.535**	**0.568**	-0.108	0.229	-0.038	0.036	-0.165	**0.598**	**0.714**	**0.966**	----	0.000	0.998	0.445	0.438	0.779
DOC	**0.366**	**-0.629**	**0.397**	0.159	0.239	0.044	0.169	-0.073	**0.725**	**0.664**	**0.677**	**0.668**	----	0.311	0.592	0.761	0.817
P_{15}	**-0.469**	-0.079	0.201	0.044	**0.508**	**0.508**	0.352	0.006	-0.043	-0.079	0.044	0.000	0.192	----	0.000	0.000	0.000
UI_{15}	**0.602**	0.086	-0.225	-0.126	**-0.517**	**-0.435**	-0.163	-0.223	0.048	0.238	0.092	0.145	0.102	**-0.756**	----	0.000	0.000
R_{15}	**0.741**	0.041	-0.217	0.010	**-0.395**	**-0.459**	-0.338	-0.262	0.177	0.289	0.158	0.147	0.058	**-0.713**	**0.793**	----	0.000
F_{15}	**0.701**	0.011	-0.216	0.082	-0.352	**-0.448**	-0.308	-0.219	0.215	0.289	0.096	0.053	0.044	**-0.627**	**0.659**	**0.950**	----

Table 2S. Partition of variance components (%) for the resemblance matrices of environmental variables (euclidean distance) and phytoplankton taxa (Bray-Curstis distance) measured at the sampling stations. The results were obtained by a sequential PERMANOVA with station as a fixed factor and sampling month as a random factor. Mean squares (MS) were computed from Type I sum of squares (Anderson et al. 2008). Variable names as in Table 1S. df: degrees of freedom, Pseudo-F: variance ratio, P: significance of Pseudo-F.

		resemblance matrix	
term	factor	environmental	phytoplankton
MS	station	13237.00	106.43
	month	3413.20	31.91
	error	2287.00	7.66
df	station	2	2
	month	9	9
	error	18	18
Pseudo-F	station	13.89	5.79
	month	4.17	1.49
P	station	0.001	0.001
	month	0.001	0.003
% Variance	station	29.14	38.55
	month	9.99	31.55
	error	60.86	29.90

Table 3S. Mean, minimum (Min) and maximum (Max) abundance (cells mL^{-1}) of the phytoplankton species or groups identified during the study at the diferent stations. Taxonomic information updated following the World Register of Marine Species (http://www.marinespecies.org/, accessed April, 2015).

Species or group	Authority	Functional group	St. 1			St. 5			St. 10		
			Mean	Min	Max	Mean	Min	Max	Mean	Min	Max
Actinoptychus senarius	(Ehrenberg) Ehrenberg, 1843	Bacillariophyceae	----	----	----	4.4	4.4	4.4	----	----	----
Anabaena spiralis	Wm.Thompson	Cyanophyceae	----	----	----	----	----	----	14,565.6	14,565.6	14,565.6
Ankistrodesmus spp.	Corda, 1838	Chlorophyceae	----	----	----	----	----	----	238.9	15.4	462.4
Ankistrodesmus falcatus	(Corda) Ralfs, 1848	Chlorophyceae	----	----	----	42.2	4.4	80.0	140.4	57.8	228.8
Aphanocapsa spp.	Nägeli, 1849	Cyanophyceae	----	----	----	----	----	----	19,360.0	19,360.0	19,360.0
Asterionella formosa	Hassall, 1850	Bacillariophyceae	----	----	----	44.4	28.8	60.0	98.4	43.5	160.0
Asterionellopsis glacialis	(Castracane) Round, 1990	Bacillariophyceae	8.3	2.2	17.6	----	----	----	----	----	----
Aulacoseira granulata	(Ehrenberg) Simonsen, 1979	Bacillariophyceae	----	----	----	9.9	8.8	11.0	108.1	26.4	319.0
Cerataulina pelagica	(Cleve) Hendey, 1937	Bacillariophyceae	4.4	4.4	4.4	----	----	----	----	----	----
Chaetoceros affinis	Lauder, 1864	Bacillariophyceae	1,095.6	44.0	3,872.0	----	----	----	----	----	----
Chaetoceros atlanticus	P.T. Cleve, 1873	Bacillariophyceae	2.0	2.0	2.0	----	----	----	----	----	----
Chaetoceros compressus	Lauder, 1864	Bacillariophyceae	44.0	44.0	44.0	----	----	----	----	----	----
Chaetoceros curvisetus	Cleve, 1889	Bacillariophyceae	5.5	4.4	6.6	----	----	----	----	----	----
Chaetoceros debilis	Cleve, 1894	Bacillariophyceae	17.6	17.6	17.6	----	----	----	----	----	----
Chaetoceros diadema	(Ehrenberg) Gran, 1897	Bacillariophyceae	22.0	22.0	22.0	----	----	----	----	----	----
Chaetoceros didymus	Ehrenberg, 1845	Bacillariophyceae	10.3	1.0	26.4	8.8	8.8	8.8	----	----	----
Chaetoceros simplex	Ostenfeld, 1901	Bacillariophyceae	8.8	8.8	8.8	----	----	----	----	----	----
Chaetoceros socialis	H.S.Lauder, 1864	Bacillariophyceae	752.5	13.6	3,326.4	----	----	----	----	----	----
Chaetoceros spp.	Ehrenberg, 1844	Bacillariophyceae	69.8	2.0	237.6	61.1	35.2	87.0	----	----	----

Species or group	Authority	Functional group	St. 1 Mean	Min	Max	St. 5 Mean	Min	Max	St. 10 Mean	Min	Max
Chlorophyceae (undetermined)		Chlorophyceae	----	----	----	----	----	----	126.5	70.4	203.0
Chroococcus spp.	Nägeli, 1849	Cyanophyceae	----	----	----	2,281,786.7	20,960.0	6,736,400.0	1,104,605.3	46,816.0	2,475,000.0
Cocconeis scutellum	Ehrenberg, 1838	Bacillariophyceae	2.2	1.0	3.3	----	----	----	----	----	----
Corymbellus sp.	J.C.Green, 1976	Prymnesiophyceae	35.2	35.2	35.2	----	----	----	----	----	----
Cryptophyceae >20µm	Fritsch, 1927	Cryptophyceae	----	----	----	446.0	88.0	1,440.0	456.1	86.7	1,271.6
Cryptophyceae 10-20µm	Fritsch, 1927	Cryptophyceae	45.6	8.8	114.4	82.7	17.6	160.0	508.2	160.0	800.0
Cryptophyceae <10µm	Fritsch, 1927	Cryptophyceae	----	----	----	203.6	28.9	501.6	1,264.8	52.8	5,202.0
Cystodinium sp.	Klebs, 1912	Dinophyceae	----	----	----	2.2	2.2	2.2	1,329.4	1,329.4	1,329.4
Desmodesmus communis	(E.H.Hegewald) E.H.Hegewald	Chlorophyceae	----	----	----	28.8	28.8	28.8	127.9	8.8	240.0
Detonula pumila	(Castracane) Gran, 1900	Bacillariophyceae	13.2	13.2	13.2	8.8	8.8	8.8	----	----	----
Diatoms < 20 µm		Bacillariophyceae	----	----	----	777.0	777.0	777.0	----	----	----
Diatoms centric <20 µm		Bacillariophyceae	13.2	13.2	13.2	----	----	----	----	----	----
Diatoms centric >20 µm		Bacillariophyceae	12.6	2.2	27.2	----	----	----	14.5	14.5	14.5
Diatoms pennate <10 µm		Bacillariophyceae	17.6	17.6	17.6	----	----	----	28.9	28.9	28.9
Diatoms pennate <20 µm		Bacillariophyceae	14.9	1.7	27.2	27.6	13.2	35.2	----	----	----
Diatoms pennate >20 µm		Bacillariophyceae	4.9	1.0	13.2	70.5	4.4	240.0	44.5	29.0	60.0
Dinobryon sertularia	Ehrenberg, 1834	Chrysophyceae	----	----	----	----	----	----	1,620.0	1,620.0	1,620.0
Dinoflagellates < 20 µm		Dinophyceae	38.7	8.8	96.8	152.4	26.4	387.2	64.4	17.6	160.0
Dinoflagellates > 20 µm		Dinophyceae	7.0	1.0	16.5	155.5	6.6	642.4	31.9	6.6	60.0

Species or group	Authority	Functional group	St. 1 Mean	Min	Max	St. 5 Mean	Min	Max	St. 10 Mean	Min	Max
Dinophysis acuminata	Claparède & Lachmann, 1859	Dinophyceae	4.4	4.4	4.4	600.0	600.0	600.0	----	----	----
Diploneis didyma	(Ehrenberg) Ehrenberg, 1845	Bacillariophyceae	----	----	----	8.8	8.8	8.8	----	----	----
Diplopsalis spp.	Bergh, 1881	Dinophyceae	2.2	2.2	2.2	----	----	----	----	----	----
Distephanus speculum	(Ehrenberg) Haeckel, 1887	Dictyochophyceae	2.5	1.0	4.4	----	----	----	----	----	----
Eucampia zodiacus	Ehrenberg, 1839	Bacillariophyceae	18.7	2.2	35.2	2.2	2.2	2.2	----	----	----
Eunotia spp.	Ehrenberg, 1837	Bacillariophyceae	----	----	----	----	----	----	7,360.0	7,360.0	7,360.0
Eutreptia sp.	Perty, 1852	Euglenoidea	2.2	2.2	2.2	362.8	52.8	1,135.2	----	----	----
Fragilaria crotonensis	Kitton, 1869	Bacillariophyceae	8.0	8.0	8.0	3,527.0	35.2	12,720.0	7,010.0	43.2	40,691.2
Fragilaria spp.	Lyngbye, 1819	Bacillariophyceae	----	----	----	----	----	----	14.5	14.5	14.5
Goniochloris mutica	(A.Braun) Fott, 1960	Xanthophyceae	----	----	----	----	----	----	86.7	86.7	86.7
Gonyaulax spinifera	(Claparède & Lachmann) Diesing, 1866	Dinophyceae	2.2	2.2	2.2	----	----	----	----	----	----
Guinardia delicatula	(Cleve) Hasle, 1997	Bacillariophyceae	54.1	6.6	220.0	----	----	----	----	----	----
Gyrodinium britannicum	Kofoid & Swezy, 1921	Dinophyceae	2.2	2.2	2.2	----	----	----	----	----	----
Gyrodinium spirale	(Bergh) Kofoid & Swezy, 1921	Dinophyceae	4.1	2.2	8.8	70.0	60.0	80.0	----	----	----
Gyrodinium spp.	Kofoid & Swezy, 1921	Dinophyceae	----	----	----	8.8	8.8	8.8	----	----	----
Gyrosigma fasciola	(Ehrenberg) J.W.Griffith & Henfrey, 1856	Bacillariophyceae	----	----	----	3.3	2.2	4.4	----	----	----

Species or group	Authority	Functional group	St. 1 Mean	St. 1 Min	St. 1 Max	St. 5 Mean	St. 5 Min	St. 5 Max	St. 10 Mean	St. 10 Min	St. 10 Max
Gyrosigma sp.	Hassall, 1845	Bacillariophyceae	----	----	----	4.4	4.4	4.4	----	----	----
Heterocapsa niei	(Loeblich III) Morrill & Loeblich III, 1981	Dinophyceae	18.3	13.6	23.8	52.8	52.8	52.8	----	----	----
Katodinium glaucum	(Lebour) Loeblich III, 1965	Dinophyceae	8.8	4.4	13.2	----	----	----	----	----	----
Katodinium spp.	Fott, 1857	Dinophyceae	6.8	6.8	6.8	----	----	----	----	----	----
Kirchneriella sp.	Schmidle, 1893	Chlorophyceae	----	----	----	----	----	----	115.6	115.6	115.6
Lauderia annulata	Cleve, 1873	Bacillariophyceae	4.0	1.0	6.6	----	----	----	----	----	----
Leptocylindrus danicus	Cleve, 1889	Bacillariophyceae	52.8	26.4	79.2	13.2	13.2	13.2	----	----	----
Leptocylindrus minimus	Gran, 1915	Bacillariophyceae	25.3	8.8	52.8	----	----	----	----	----	----
Lingulodinium polyedrum	(F.Stein) J.D.Dodge, 1989	Dinophyceae	17.2	1.0	44.0	----	----	----	----	----	----
Lyngbya sp.	C.Agardh ex Gomont, 1892	Cyanophyceae	----	----	----	----	----	----	324.5	289.0	360.0
Melosira lineata	(Dillwyn) C.Agardh, 1824	Bacillariophyceae	----	----	----	8.8	8.8	8.8	----	----	----
Melosira nummuloides	C.Agardh, 1824	Bacillariophyceae	----	----	----	118.1	6.6	360.0	----	----	----
Melosira spp.	C.Agardh, 1824	Bacillariophyceae	----	----	----	----	----	----	101.5	101.5	101.5
Micracanthodinium spp.	Deflandre, 1937	Dinophyceae	2.8	1.7	4.4	57.8	57.8	57.8	----	----	----
Mycrocystis spp.	Lemmermann, 1907	Cyanophyceae	----	----	----	----	----	----	2,880.0	2,880.0	2,880.0
Navicula cancellata	Donkin, 1872	Bacillariophyceae	----	----	----	4.4	4.4	4.4	----	----	----
Navicula spp.	Bory de Saint-Vincent, 1822	Bacillariophyceae	----	----	----	60.0	60.0	60.0	----	----	----
Navicula transitans	Cleve, 1883	Bacillariophyceae	6.4	1.7	8.8	62.0	2.2	180.0	8.8	8.8	8.8

Species or group	Authority	Functional group	St. 1 Mean	Min	Max	St. 5 Mean	Min	Max	St. 10 Mean	Min	Max
Neoceratium furca	(Ehrenberg) F.Gomez, D.Moreira & P.Lopez-Garcia, 2010	Dinophyceae	2.6	2.2	3.3	----	----	----	----	----	----
Neoceratium fusus	(Ehrenberg) F.Gomez, D.Moreira & P.Lopez-Garcia, 2010	Dinophyceae	2.2	2.2	2.2	----	----	----	----	----	----
Nitzschia bicapitata	Cleve, 1901	Bacillariophyceae	8.8	8.8	8.8	----	----	----	----	----	----
Nitzschia longissima	(Brébisson) Ralfs, 1861	Bacillariophyceae	6.5	2.2	22.0	18.4	4.4	60.0	----	----	----
Nitzschia longissima (small)	(Brébisson) Ralfs, 1861	Bacillariophyceae	36.9	2.2	118.8	30.0	4.4	72.5	----	----	----
Nitzschia spp. (epiphytic)	Hassall, 1845	Bacillariophyceae	10.3	4.4	17.6	----	----	----	----	----	----
Nitzschia tryblionella	Hantzsch, 1860	Bacillariophyceae	1.1	1.1	1.1	4.4	4.4	4.4	----	----	----
Odontella mobiliensis	(J.W.Bailey) Grunow, 1884	Bacillariophyceae	22.0	22.0	22.0	----	----	----	----	----	----
Oocystis spp.	Nägeli ex A.Braun, 1855	Chlorophyceae	----	----	----	----	----	----	17.6	17.6	17.6
Oscillatoria spp.	Vaucher ex Gomont, 1892	Cyanophyceae	20.4	20.4	20.4	----	----	----	----	----	----
Pediastrum duplex	Meyen, 1829	Chlorophyceae	----	----	----	----	----	----	538.7	70.4	1,200.0
Peridinium aciculiferum	Lemmermann, 1900	Dinophyceae	----	----	----	551.0	551.0	551.0	----	----	----
Phacus spp.	Dujardin, 1841	Euglenoidea	----	----	----	14.5	14.5	14.5	----	----	----
Phaeocystis pouchetii	(Hariot) Lagerheim, 1896	Prymnesiophyceae	15.0	15.0	15.0	----	----	----	----	----	----

Species or group	Authority	Functional group	St. 1 Mean	Min	Max	St. 5 Mean	Min	Max	St. 10 Mean	Min	Max
Prorocentrum balticum	(Lohmann) Loeblich, 1970	Dinophyceae	6.5	1.7	17.6	----	----	----	----	----	----
Prorocentrum cordatum	(Ostenfeld) Dodge, 1975	Dinophyceae	4.4	4.4	4.4	----	----	----	----	----	----
Prorocentrum micans	Ehrenberg, 1834	Dinophyceae	1.9	1.0	2.2	----	----	----	----	----	----
Protoperidinium bipes	(Paulsen) Balech, 1974	Dinophyceae	8.7	8.5	8.8	8.8	8.8	8.8	----	----	----
Protoperidinium depressum	(Bailey) Balech, 1974	Dinophyceae	2.2	2.2	2.2	----	----	----	----	----	----
Protoperidinium diabolum	(Cleve) Balech, 1974	Dinophyceae	2.2	2.2	2.2	----	----	----	----	----	----
Protoperidinium divergens	(Ehrenberg) Balech, 1974	Dinophyceae	1.6	1.0	2.2	----	----	----	----	----	----
Protoperidinium leonis	(Pavillard) Balech, 1974	Dinophyceae	2.2	2.2	2.2	----	----	----	----	----	----
Protoperidinium oblongum	(Aurivillius) Parke & Dodge, 1976	Dinophyceae	3.3	3.3	3.3	----	----	----	----	----	----
Protoperidinium ovatum	Pouchet, 1883	Dinophyceae	2.2	2.2	2.2	----	----	----	----	----	----
Protoperidinium spp.	Bergh, 1882	Dinophyceae	----	----	----	152.8	4.4	540.0	----	----	----
Protoperidinium steinii	(Jörgensen) Balech, 1974	Dinophyceae	7.3	4.4	8.8	----	----	----	----	----	----
Pseudo-nitzschia delicatissima	(Cleve) Heiden, 1928	Bacillariophyceae	20.8	2.0	39.6	----	----	----	----	----	----
Pseudo-nitzschia pungens	(Grunow ex Cleve) G.R.Hasle, 1993	Bacillariophyceae	253.2	3.3	1,232.0	4.4	4.4	4.4	----	----	----
Pseudo-nitzschia spp.	H.Peragallo, 1900	Bacillariophyceae	----	----	----	120.0	120.0	120.0	----	----	----
Rhizosolenia imbricata	Brightwell, 1858	Bacillariophyceae	2.2	2.2	2.2	----	----	----	----	----	----
Rhoicosphenia abbreviata	(C.Agardh) Lange-Bertalot, 1980	Bacillariophyceae	----	----	----	4.4	4.4	4.4	----	----	----
Scenedesmus ecornis	(Ehrenberg) Chodat	Chlorophyceae	----	----	----	17.6	17.6	17.6	23.3	17.6	29.0

Species or group	Authority	Functional group	St. 1 Mean	St. 1 Min	St. 1 Max	St. 5 Mean	St. 5 Min	St. 5 Max	St. 10 Mean	St. 10 Min	St. 10 Max
Scenedesmus spp.	Meyen, 1829	Chlorophyceae	----	----	----	----	----	----	480.0	480.0	480.0
Scrippsiella trochoidea	(Stein) Balech ex Loeblich III, 1965	Dinophyceae	20.1	4.4	57.2	8.8	8.8	8.8	----	----	----
Selenastrum spp.	Reinsch, 1867	Chlorophyceae	----	----	----	----	----	----	57.8	57.8	57.8
Shionodiscus oestrupii	(Ostenfeld) A.J.Alverson, S.H.Kang & E.C.Theriot, 2006	Bacillariophyceae	32.6	2.0	112.2	23.3	17.6	29.0	103.9	26.4	144.5
Skeletonema costatum	(Greville) Cleve, 1873	Bacillariophyceae	22.0	17.6	26.4	----	----	----	----	----	----
Solenicola setigera	Pavillard, 1916	Flagellates	215.6	215.6	215.6	----	----	----	----	----	----
Staurastrum paradoxum	Meyen ex Ralfs, 1848	Charophyta	----	----	----	----	----	----	114.4	114.4	114.4
Staurastrum spp.	Meyen ex Ralfs, 1848	Charophyta	----	----	----	----	----	----	104.5	28.9	180.0
Staurodesmus spp.	Teiling, 1948	Charophyta	----	----	----	----	----	----	14.5	14.5	14.5
Stenopterobia intermedia	(F.W.Lewis) Van Heurck, 1896	Bacillariophyceae	----	----	----	2.2	2.2	2.2	----	----	----
Stephanopyxis turris	(Greville) Ralfs, 1861	Bacillariophyceae	----	----	----	17.6	17.6	17.6	----	----	----
Striatella unipunctata	(Lyngbye) C.Agardh, 1832	Bacillariophyceae	4.4	4.4	4.4	----	----	----	----	----	----
Surirella spp.	Turpin, 1828	Bacillariophyceae	----	----	----	60.0	60.0	60.0	182.3	144.5	220.0
Synura uvella	Ehrenberg, 1834	Synurophyceae	----	----	----	----	----	----	38,226.0	1,452.0	75,000.0
Tetraedron caudatum	(Corda) Hansgirg, 1888	Chlorophyceae	----	----	----	----	----	----	28.9	28.9	28.9
Tetraedron minutum	(A.Braun) Hansgirg	Chlorophyceae	----	----	----	----	----	----	68.7	28.9	115.6
Tetraedron triangulare	Korshikov	Chlorophyceae	----	----	----	----	----	----	260.1	260.1	260.1
Tetraspora limnetica	West & G.S.West, 1912	Chlorophyceae	----	----	----	52.8	52.8	52.8	357.7	174.0	667.0

Species or group	Authority	Functional group	St. 1			St. 5			St. 10		
			Mean	Min	Max	Mean	Min	Max	Mean	Min	Max
Thalassionema nitzschioides	(Grunow) Mereschkowsky, 1902	Bacillariophyceae	44.0	17.6	70.4	----	----	----	----	----	----
Thalassiosira angulata	(W.Gregory) Hasle, 1978	Bacillariophyceae	4.0	4.0	4.0	----	----	----	----	----	----
Thalassiosira antarctica	Comber, 1896	Bacillariophyceae	7.6	2.0	13.2	----	----	----	----	----	----
Thalassiosira levanderi	van Goor, 1924	Bacillariophyceae	5.1	1.7	8.4	----	----	----	----	----	----
Thalassiosira spp.	Cleve, 1873	Bacillariophyceae	2.2	2.2	2.2	----	----	----	----	----	----
Torodinium robustum	Kofoid & Swezy, 1921	Dinophyceae	4.4	4.4	4.4	----	----	----	----	----	----
Torodinium teredo	(Pouchet) Kofoid & Swezy, 1921	Dinophyceae	----	----	----	4.4	4.4	4.4	----	----	----
Tryblionella apiculata	Gregory, 1857	Bacillariophyceae	----	----	----	2.2	2.2	2.2	----	----	----

Table 4S. Parameters of functions fitted to data in Fig. 7. Function types are Michaelis-Menten (M-M): $y = a\,x/(b+x)$ or linear: $y = ax + b$. The Akaike Information Criterion are given for the M-M (AIC_{MM}) and linear fits (AIC_{linear}). Pearson's r correlation and significance of linear fit (P_{linear}) are also given for comparison. Variable names for x and y as in Table 1S. Autocorrelation was low for all variables (Fig. 2S).

Stations	*y*	*x*	function type	*a*	*b*	AIC_{MM}	AIC_{linear}	Pearson's r	P_{linear}
St. 1 & St. 5	shared taxa	F_{15}	M-M	1.920	-2.758	30.605	49.743	-0.517	0.126
St. 5 & St. 10	shared taxa	F_{15}	M-M	3.643	2.453	15.760	16.252	0.428	0.225
St. 1 & St. 5	β-diversity	F_{15}	M-M	0.867	0.978	5.770	5.791	0.498	0.144
St. 5 & St. 10	β-diversity	F_{15}	M-M	0.634	-0.914	5.772	5.770	-0.726	0.017
St. 1 & St. 5	shared taxa	P_{15}	M-M	3.150	-1.034	55.033	62.494	-0.235	0.513
St. 5 & St. 10	shared taxa	P_{15}	M-M	3.685	3.228	15.989	14.433	0.569	0.086
St. 1 & St. 5	β-diversity	P_{15}	M-M	0.812	0.366	5.804	5.816	0.079	0.827
St. 5 & St. 10	β-diversity	P_{15}	M-M	0.690	-0.348	5.815	5.828	-0.207	0.493
St. 1 & St. 5	shared taxa	UI_{15}	Linear	0.027	3.858	---	60.454	0.298	0.402
St. 5 & St. 10	shared taxa	UI_{15}	Linear	-0.033	2.705	---	10.466	-0.795	0.006
St. 1 & St. 5	β-diversity	UI_{15}	Linear	-0.001	0.777	---	5.815	-0.119	0.743
St. 5 & St. 10	β-diversity	UI_{15}	Linear	0.002	0.734	---	5.802	0.505	0.136

Table 5S. Results of PERMANOVA+ tests (Anderson et al. 2008) on the number of shared taxa and β-diversity between St. 1 and 5, and between St. 1 and 10, on the effects of upwelling and river flow. Each sampling date was classified as upwelling (i.e. positive values of UI) or downwelling (i.e. negative values of UI), and as high flow (≥20 hm^3 per15 d) or low flow (<20 hm^3 per 15 d). Raw data were standardized and resemblance matrices were computed using the euclidean distance. df: degrees of freedom, SS: sum of squares, MS: mean squares, Pseudo-F: MS ratio, P: significance. Autocorrelation was low for all variables (Fig. 2S).

Index	Source	df	SS	MS	Pseudo-F	P
Shared taxa	Upwelling	1	1894.90	1894.90	6.237	0.023
	River flow	1	1933.10	1933.10	6.363	0.031
	interaction	1	478.28	478.28	1.574	0.278
	Residuals	6	1823.00	303.83		
	Total	9	6129.40			
β-diversity	Upwelling	1	91.48	91.48	2.423	0.170
	River flow	1	114.93	114.93	3.044	0.124
	interaction	1	3.50	3.50	0.093	0.773
	Residuals	6	226.53	37.76		
	Total	9	436.44			

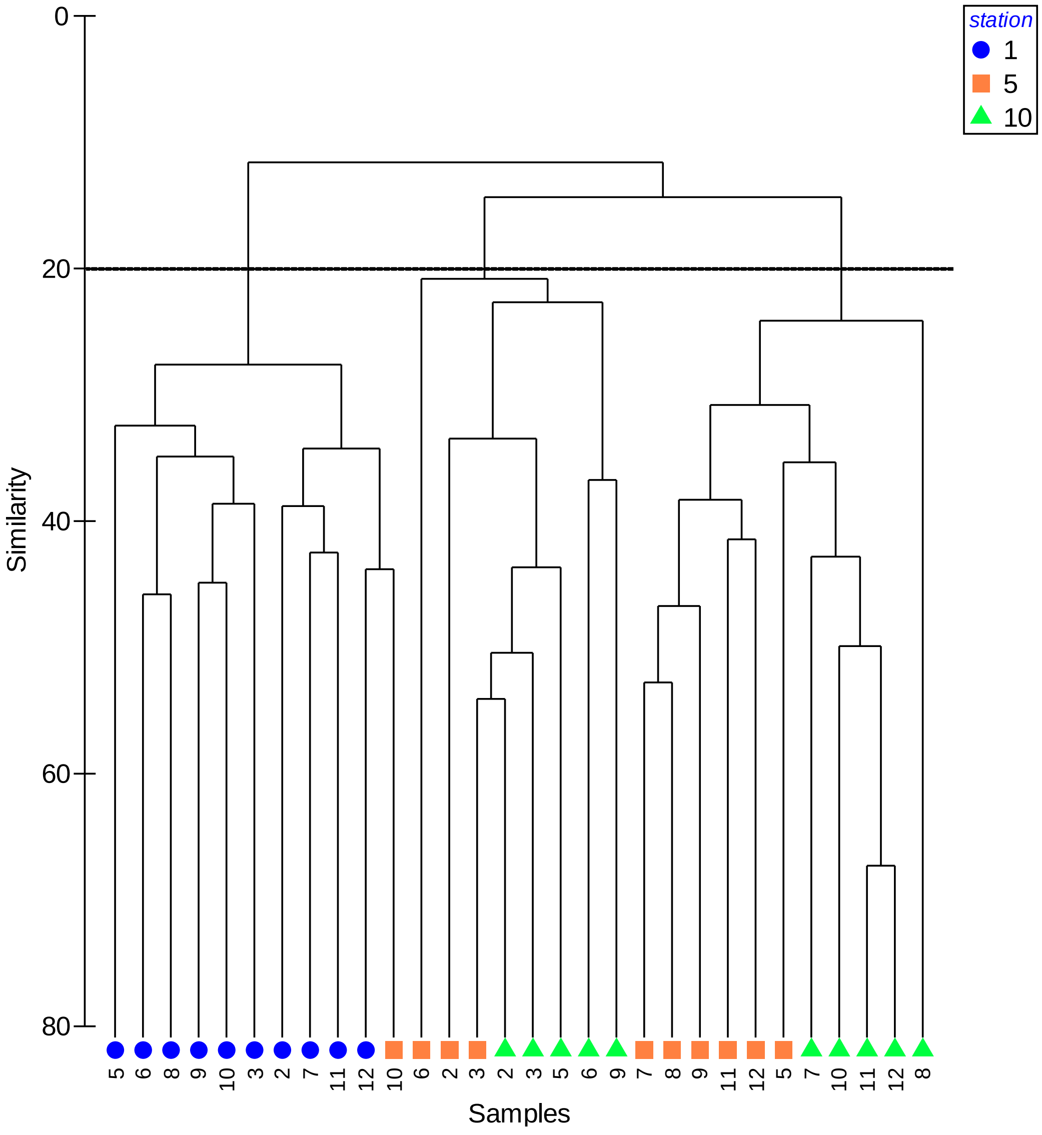

Fig. 1S. Dendrogram of samples clustered using log-transformed species abundance. Numbers indicate the sampling month and the different stations are indicated by colours. Group-average cluster on Bray-Curtis similarity matrix. The similarity level of 20% (dotted horizontal line) separates the groups indicated in the MDS plot (Fig. 6).

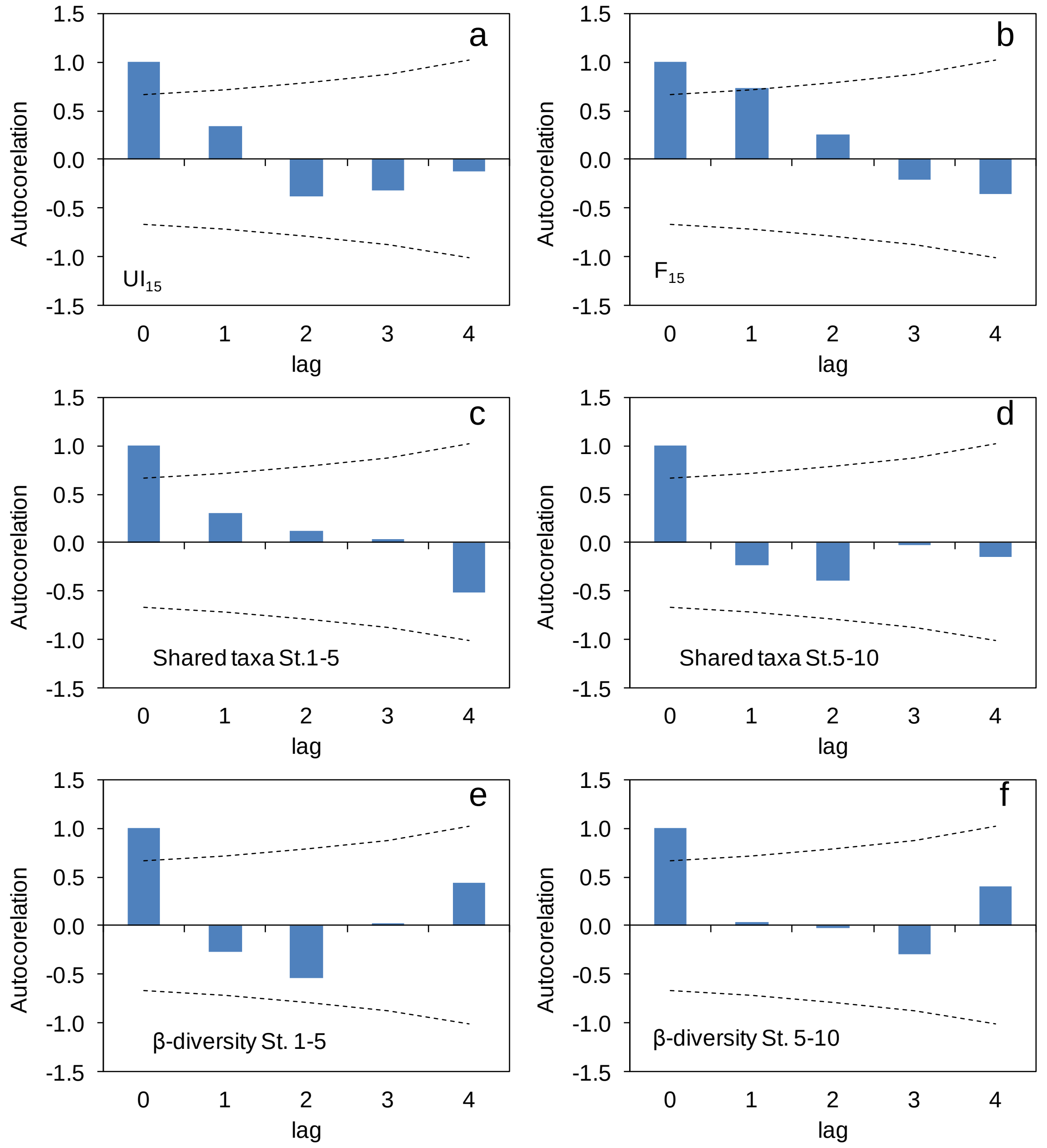

Fig. 2S. Autocorrelation of variables employed in the regression analysis of Fig. 7 and Table 4S. a) UI_{15}: accumulated Ekman transport during 15 days prior to sampling; b) F_{15}: accumulated river flow during 15 days prior to sampling; c) number of taxa shared between St. 1 and 5; d) number of taxa shared between St. 5 and 10. e) β-diversity for St. 1 and 5; f) β-diversity for St. 5 and 10. Confidence limits (95%) were indicated with dashed lines.
